# Sequence type 17 is a predictor of subsequent bacteremia in vancomycin-resistant *Enterococcus faecium-*colonized patients: a retrospective cohort study

**DOI:** 10.1186/s13756-021-00980-1

**Published:** 2021-07-22

**Authors:** Si-Ho Kim, Sun Young Cho, Hye Mee Kim, Kyungmin Huh, Cheol-In Kang, Kyong Ran Peck, Doo Ryeon Chung

**Affiliations:** 1 Division of Infectious Diseases, Samsung Medical Center, Sungkyunkwan University School of Medicine, 81 Irwon-ro, Gangnam-gu, Seoul, 06351 South Korea; 2 Center for Infection Prevention and Control, Samsung Medical Center, Seoul, Korea; 3 Asia Pacific Foundation for Infectious Diseases, Seoul, Korea; 4 Present Address: Division of Infectious Diseases, Samsung Changwon Hospital, Sungkyunkwan University School of Medicine, Changwon, Korea

**Keywords:** Microbial drug resistance, Colonization, Risk factors, Genotype, Multilocus sequence typing

## Abstract

**Background:**

Sequence type (ST) 17 vancomycin-resistant *Enterococcus faecium* (VREF) is frequently isolated in nosocomial settings. The aim of this study was to identify whether ST17 contributes to subsequent bacteremia more often than other STs among hospitalized patients carrying VREF.

**Methods:**

A retrospective cohort study was conducted in patients carrying ST17 VREF and those with non-ST17 VREF. Rectal screening according to hospital policy was used to identify patients with VREF. Subsequent VREF bacteremia events within a year of detection of colonization were recorded. Cox regression analysis was used to adjust the covariates involved in determining the association between ST17 and subsequent bacteremia events.

**Results:**

The cohorts comprised 52 patients with ST17 and 169 patients with non-ST17 VREF. One-year VREF bacteremia-free rates were 85.9% and 90.2%, respectively. In multivariate analysis, ST17 was associated with subsequent bacteremia at an adjusted hazard risk (aHR) of 4.02 (95% confidence interval [CI], 1.32–12.29). Liver transplantation (aHR, 40.08; 95% CI, 4.87–329.76) and hematologic malignancy (aHR, 20.97; 95% CI, 4.87–87.82) were also significant. All cases of subsequent bacteremia in ST17 VREF carriers were caused by ST17; however, subsequent bacteremia in non-ST17 carriers was often caused by ST17 or another ST variant.

**Conclusions:**

A specific genotype, ST17 is a predictor of subsequent bacteremia in hospitalized patients carrying VREF. Patients with a hematologic malignancy and those receiving a liver transplant are also at high risk. More targeted strategies may be needed to prevent VREF infection in hospitals.

**Supplementary Information:**

The online version contains supplementary material available at 10.1186/s13756-021-00980-1.

## Background

Vancomycin-resistant *Enterococcus faecium* (VREF) is an emerging pathogen associated with healthcare-associated infections [1]. Due to limited availability of effective antibiotics, the World Health Organization added vancomycin-resistant *Enterococcus* (VRE) to a global priority list for research and development of antibiotic-resistant bacteria [2]. *E. faecium* linked to healthcare-associated infection has shown high rates of vancomycin resistance in many countries, including Korea and the US [3, 4]. A meta-analysis revealed that VRE infections are associated with increased mortality rates and treatment costs [5].

Patients carrying VREF can develop subsequent bacteremia [6, 7], the risk factors for which reportedly include use of vancomycin, long-term antibiotic use, prolonged hospital stays, previous invasive procedures, additional body-site infections other than blood, transfers from a long-term care facility, diabetes mellitus, and acute kidney injuries [8–12]. The relationship between the risk of subsequent bacteremia among VREF carriers and the VREF genotype is not well documented. Clonal complex (CC) 17 has been identified as a hospital-adopted VREF clone [13]. In particular, sequence type (ST) 17, which is considered a predicted founder of CC17, has been isolated frequently in nosocomial settings. ST17 has a close relationship with virulence determinants in *Enterococcus* [14], and there have been several reports of in-hospital VREF outbreaks associated with ST17 [15–17]. Here, we describe the results of a retrospective cohort study of whether rectal carriage of ST17 VREF contributes to the development of subsequent bacteremia more often than do other STs.

## Methods

### Study population

This study involved VREF-colonized patients from March 2014 to February 2015 at the Samsung Medical Center, Seoul, Korea, a large tertiary referral hospital at which more than 70% of patients were referred from other regions across the country. VREF rectal screenings were performed according to infection prevention policy and protocols of the hospital. Patients transferred to the hospital from other medical or long-term care facilities and patients transferred from a general ward to intensive care units (ICUs) were subjected to rectal VRE screening.

### Study design

Two cohorts of patients carrying VREF were enrolled: those colonized with ST17 VREF and those with non-ST17 VREF. Exclusion criteria were as follows: 1) patients age under 18, 2) patients having VREF bacteremia preceding or on the same day of detection of rectal VREF colonization, and 3) Patients who died on the day of detection of rectal colonization. Subsequent VREF bacteremia events within one year of detection of colonization were recorded. All patients were observed for one year after colonization or observed until death or loss to follow-up. Clinically significant VREF bacteremia was defined as either the isolation of VREF from two or more separate blood samples, or the isolation of VREF from a single blood sample in patients with clinical symptoms and a concomitant infection [18]. Overall 30-day and 1-year survival rates were compared between VREF bacteremia group and the group of patients who did not develop VREF bacteremia.

Clinical information was reviewed during the 1-year follow-up period using electronic medical records, and data were collected on age; sex; body mass index; underlying diseases such as decompensated liver cirrhosis, severe acute kidney injury requiring renal-replacement therapy, diabetes mellitus, solid cancer, and hematologic malignancy; history of liver transplantation; neutropenia, Charlson comorbidity index [19], ICU stays; presence of central venous catheter; intubation; intra-abdominal surgery; parenteral nutrition; and specific antibiotic use prior to the onset of subsequent VREF bacteremia. The study was approved by the Institutional Review Board of the Samsung Medical Center.

### Microbiological methods

Genomic DNA of the isolates was extracted using a G-spin Genomic DNA extraction kit (iNtRON, Korea) according to the manufacturer’s instructions. Multilocus sequence typing (MLST) of seven selected housekeeping loci (*adk, atpA, ddl, gdh, gyd, purK*, and *pstS*) with polymerase chain reaction (PCR) amplification was used for genotyping of VREF isolates [20]. The e-BURST algorithm was used to analyze the relatedness of each VREF isolate ST [21]. The presence of virulence genes *esp* and *hyl* was detected by PCR [22, 23], and confirmed by sequencing [24].

In cases in which both blood and rectal VREF isolates showed an identical ST, pulsed field gel electrophoresis (PFGE) was conducted to determine the clonal association. For PFGE, bacterial DNA was digested with the *Sma* I restriction enzyme (TaKaRa Bio Inc., Shiga, Japan) and separated by electrophoresis using a CHEF DR II system (Bio-Rad Laboratories, Hercules, CA, USA). The PFGE patterns were analyzed using Gel Compar II software (Applied Maths, Kortrijk, Belgium). Potential clonal relatedness was determined at a ≥ 80% level of similarity [25].

### Statistical methods

All statistical analyses were performed in SPSS 23.0 for Window (IBM Corp., Armonk, NY, 2015) and Stata 15.1 (StataCorp., College Station, TX, USA). Power analysis for two-sample comparison of survival analysis was implemented with fixed sample size and hazard difference [26]. A Student’s t-test or Mann–Whitney U test was used to compare continuous variables and a chi-square test or Fisher’s exact test was used to compare categorical variables. Time to development of subsequent bacteremia during the 1-year follow-up period was calculated using the Kaplan–Meier method and cohort groups were compared using a log-rank test.

Two statistical models were used to adjust the covariates to determine the association between ST17 and the development of subsequent bacteremia. In model 1, each variate was compared between ST17 and non-ST17 cohorts. Variables with a *P* value < 0.15 in the univariate analysis were then included in a multivariate Cox regression model to calculate an adjusted hazard ratio (aHR) for subsequent bacteremia. Model 2 analysis evaluated other risk factors for developing VREF bacteremia. After all variates were analyzed using univariate Cox regression to calculate the hazard ratio for subsequent bacteremia, variables with a statistical significance in univariate analysis and with a probable clinical meaning were selected in multivariable Cox regression. All *P* values were two-tailed, and values < 0.05 were considered statistically significant.

## Results

### Study population

Among 254 VREF carriers identified during the study period, 221 were enrolled in the study (Additional file 1: Fig. S1). All isolates except one (99.5%) belonged to CC17. The most frequent ST was ST17 (23.5%). Among the isolates in the non-ST17 cohort, ST230 (15.8%) was the most frequent, and followed by ST981 (7.7%), ST78 (5.9%), ST192 (5.4%), ST927 (4.1%), and ST789 (3.6%) (Additional file 1: Table S1). The isolates of 98.1% of the ST17 cohort had both the *esp* and *hyl* genes, whereas 75.7% of the non-ST17 cohort had both genes (Table 1). Among underlying conditions of patients, liver transplantation was more closely associated with non-ST17.

**Table 1:** Comparison of characteristics between ST17 and non-ST17 cohorts

Variables	Total (n = 221)	Cohort					
		ST17 (n = 52)		non-ST17 (n = 169)		*P value*	
Virulence factors							
* esp*+*hyl*+	179 (80.9)	51 (98.1)		128 (75.7)		< 0.001	
Age/sex/BMI							
Age (mean ± SD)	62.3 ± 14.5	64.7 ± 14.5		61.5 ± 15.0		0.752	
Male	132 (59.7)	28 (53.8)		104 (61.5)		0.323	
BMI ≥ 25 kg/m^2^	48 (21.7)	10 (19.2)		38 (22.5)		0.619	
Underlying condition							
Decompensated LC	36 (16.3)	6 (11.5)		30 (17.8)		0.289	
AKI requiring RRT	58 (26.2)	10 (19.2)		48 (28.4)		0.189	
Diabetes mellitus	57 (25.8)	45 (26.6)		12 (23.1)		0.609	
Cancer	67 (30.3)	18 (34.6)		49 (29.0)		0.441	
Hematologic malignancy	44 (19.9)	6 (11.5)		38 (22.5)		0.084	
Liver transplantation	15 (6.8)	0 (0.0)		15 (8.9)		0.026	
Neutropenia	42 (19.0)	8 (15.4)		34 (20.1)		0.447	
CCI (Median, IQR)	3 (2–5)	3 (1.25–4)		3 (2–5)		0.773	
Invasive procedure/ICU							
Central venous catheter	124 (56.1)	95 (56.2)		29 (55.8)		0.955	
Artificial airway	57 (25.8)	15 (28.8)		42 (24.9)		0.565	
Intra-abdominal surgery	21 (9.5)	4 (7.7)		17 (10.1)		0.789	
Parenteral nutrition	114 (51.6)	25 (48.1)		89 (52.7)		0.563	
ICU Stay	157 (71.0)	35 (67.3)		122 (72.2)		0.497	
Previous antibiotic use							
Ampicillin	29 (13.1)	5 (9.6)		24 (14.2)		0.392	
Glycopeptide	121 (54.8)	33 (63.5)		88 (52.1)		0.149	
3rd- or 4th-generation cephalosporin	84 (38.0)	16 (30.8)		68 (50.2)		0.219	
Carbapenem	123 (55.7)	27 (51.9)		96 (56.8)		0.535	
Piperacillin/tazobactam	132 (59.7)	29 (55.8)		103 (60.9)		0.506	
Metronidazole	33 (14.9)	7 (13.5)		26 (15.4)		0.734	
Fluoroquinolone	68 (30.8)	12 (23.1)		56 (33.3)		0.162	
Aminoglycoside	20 (9.0)	7 (13.5)		13 (7.7)		0.266	
Linezolid	16 (7.2)	3 (5.8)		13 (7.7)		0.768	

Data presented as numbers (%) unless indicated otherwise. BMI, body-mass index; LC, liver cirrhosis; AKI, acute kidney injury; RRT, renal replacement therapy, CCI, Charlson comorbidity index; IQR, interquartile range; ICU, intensive care unit

The incidence rate of subsequent VREF bacteremia was 0.447 cases per 1000 patient-days (16 cases per 35,816 observation days). The median observation day of patients who developed VREF bacteremia (VRE-B group) was 28 (range, 3–187 days). The most frequent underlying disease in the VRE-B group was hematologic malignancy (10 of 16, 62.5%) followed by liver transplantation (3 of 16, 18.8%) (Table 2). Overall 30-day and 1-year survival rates in the VRE-B group and the group of patients who did not develop VREF bacteremia were 75.0% versus 79.9% (*P* = 0.615) and 37.5% versus 57.5% (*P* = 0.073), respectively.

**Table 2:** Characteristics of patients developing subsequent vancomycin-resistant *Enterococcus faecium* bacteremia

Patient	Sex	Age	Underlying disease	Neutro-penia	Genotype of rectal isolates				Sequence type of blood isolates	Type of infection	Time interval between rectal carriage and bacteremia (days)	Antibiotic treatment	Outcome of Hospitalization
					Vancomycin -resistance gene	Virulence factor	Sequence type	Clonal complex					
A1	M	56	Cancer	−	*van A*	*esp, hyl*	17	17	N/A	Intra-abdominal infection	139	Linezolid	Alive
A2*	F	52	Myelodysplastic syndrome	+	*van A*	*esp, hyl*	17	17	17	Intra-abdominal infection	10	Linezolid Tigecycline	Alive
A3	F	57	Leukemia	+	*van A*	*esp, hyl*	17	17	N/A	Intra-abdominal infection	33	Linezolid	Alive
A4*	M	67	Lymphoma	+	*van A*	*esp, hyl*	17	17	17	Intra-abdominal infection	7	None	Died of VREF BSI
A5*	F	62	Leukemia	+	*van A*	*esp, hyl*	17	17	17	Intra-abdominal infection	4	None	Died of VREF BSI
A6*	M	70	Cancer	−	*van A*	*esp, hyl*	17	17	17	Intra-abdominal infection	6	Linezolid Tigecycline	Died of other cause
B1*	F	69	Liver cirrhosis	−	*van A*	*hyl*	389	17	389	Primary bacteremia	22	Linezolid	Died of other cause
B2*	F	51	Lymphoma	+	*van A*	*esp, hyl*	981	17	981	Urinary tract infection	12	Linezolid	Died of other cause
B3	M	78	Lymphoma	+	*van A*	*esp, hyl*	192	17	N/A	Intra-abdominal infection	8	Linezolid	Alive
B4	F	42	Leukemia	+	*van A*	*esp,hyl*	230	17	N/A	Catheter-related infection	43	Linezolid	Died of VREF BSI
B5*	M	48	Leukemia	+	*van A*	*hyl*	252	17	252	Catheter-related infection	3	Linezolid	Died of other cause
B6	M	54	Liver transplantation	−	*van A*	*esp, hyl*	978	17	1421	Intra-abdominal infection	162	Linezolid Tigecycline	Alive
B7	F	58	Lymphoma	+	*van A*	*esp, hyl*	230	17	17	Intra-abdominal infection	103	Linezolid	Died of other cause
B8	F	55	Liver cirrhosis / transplantation	−	*van A*	*esp, hyl*	230	17	17	Intra-abdominal infection	37	None	Died of other cause
B9	M	56	Lymphoma	+	*van A*	*esp, hyl*	230	17	17	Catheter-related infection	40	Linezolid	Died of VREF BSI
B10	M	47	Liver cirrhosis / Liver transplantation	+	*van A*	*hyl*	1026	17	981	Intra-abdominal infection	187	Linezolid Tigecycline	Alive

^*^Patients who had identical ST isolates of rectal swab and blood stream infection; VREF, vancomycin-resistant *Enterococcus feacium*; BSI, bloodstream infection; LT, liver transplantation; UTI, urinary tract infection; IAI, intra-abdominal infection; MDS, myelodysplastic syndrome; CRI, catheter-related infection; LC, liver cirrhosis; N/A, not available

### ST17 as a risk factor for subsequent bacteremia

The ST17 and non-ST17 cohorts included 52 cases and 169 cases, respectively. The non-ST17 cohort included 32 isolates with single-locus variants and 77 double-locus variants of ST17, among which ST230 was most frequent (Additional file 1: Table S1 and Fig. S2).

**Fig. 1:**
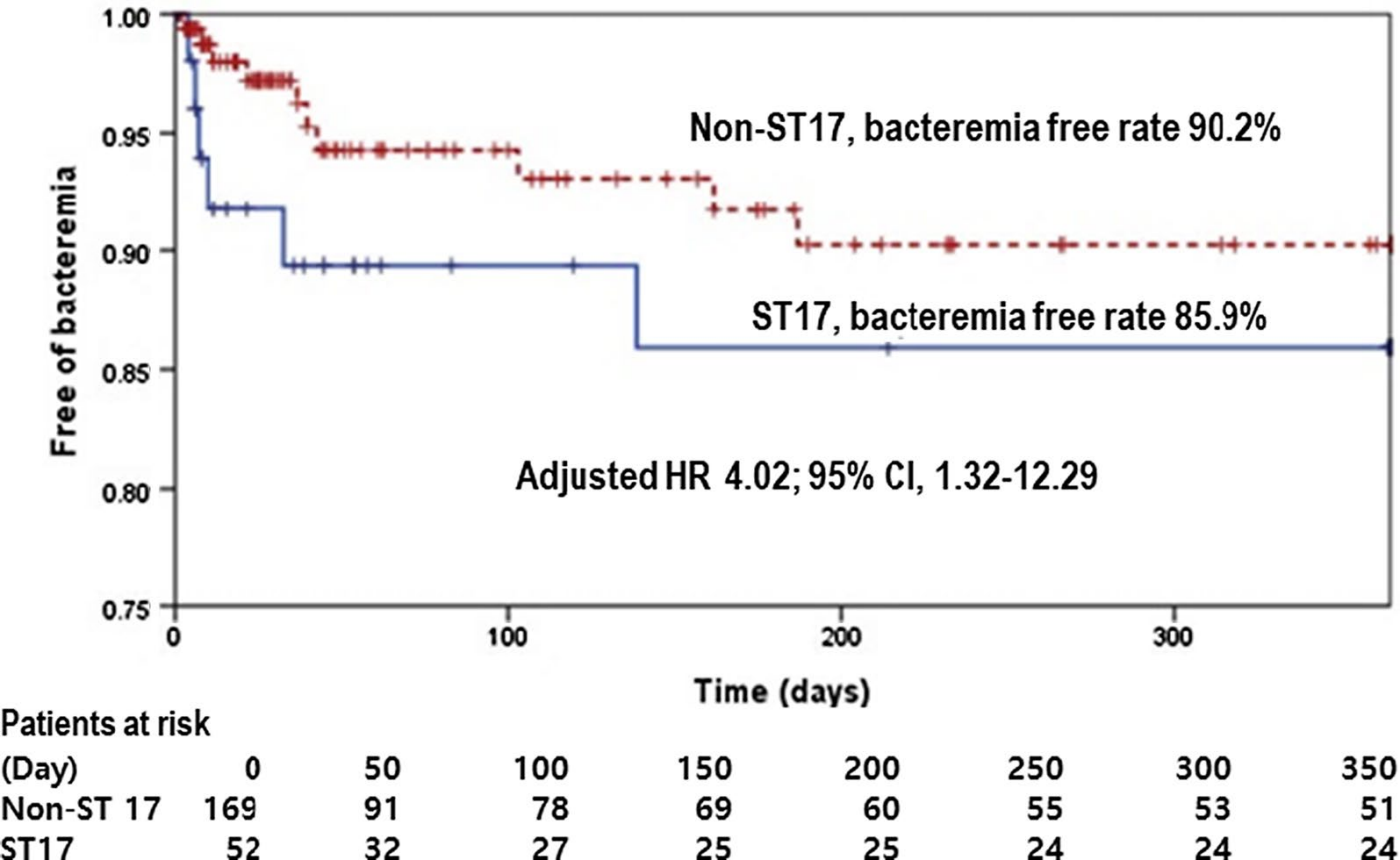
Kaplan–Meier curve of probability of free of subsequent bacteremia

Subsequent VREF bacteremia developed in 11.5% and 5.9% of ST17 and non-ST17 cohorts, respectively (*P* = 0.257). Adjusted analyses using two models showed a significant association between ST17 and subsequent VREF bacteremia. The first model analysis in which virulence factors, hematologic malignancy, liver transplantation, and previous glycopeptide use were included in multivariate analysis revealed that ST17 was significantly associated with developing subsequent VREF bacteremia (aHR, 4.02; 95% confidence interval [CI], 1.32–12.29, *P* = 0.015) (Fig. 1). The statistical power was > 0.999, which was calculated from sample size (n = 221), ST17/non-ST17 ratio 0.38 and the aHR 4.02. In the second model, in which VREF ST17, renal replacement therapy, hematologic malignancy, liver transplantation, and previous use of glycopeptide, and carbapenem were included in multivariable analysis, ST17 showed a significant association with development of subsequent VREF bacteremia (aHR, 7.14; 95% CI, 1.83–27.83; *P* = 0.005). In addition, liver transplantation (aHR, 40.08 32.65; 95% CI, 5.01–329.76 4.27–249.75; *P* = 0.001), and hematologic malignancy (aHR, 20.97; 95% CI, 5.01–87.82; *P* < 0.001) were also determined to be significant risk factors for subsequent bacteremia (Table 3).

**Table 3:** Multivariate analysis for risk factors predictive of subsequent bacteremia among hospitalized patients carrying vancomycin-resistant *Enterococcus faecium*

Variables	Total (n = 221)	Univariate analysis		Multivariable analysis	
		HR (95% CI)	*P* value	HR (95% CI)	*P* value
Virulence factors					
Sequence type 17	52 (23.5)	1.78 (0.65–4.91)	0.264	7.14 (1.83–27.83)	0.005
* esp*+*hyl*+	179 (80.9)	1.10 (0.31–3.86)	0.883		
Age/sex/BMI					
Age ≥ 60 years	132 (59.7)	0.34 (0.12–0.99)	0.048*		
Male	132 (59.7)	0.74 (0.28–1.98)	0.549		
BMI ≥ 25 kg/m^2^	48 (21.7)	0.51 (0.12–2.27)	0.379		
Underlying condition					
Decompensated LC	36 (16.3)	1.16 (0.33–4.09)	0.813		
AKI requiring RRT	38 (17.2)	3.07 (1.06–8.92)	0.039		
Diabetes mellitus	57 (25.8)	0.74 (0.21–2.59)	0.636		
Metastatic cancer	67 (30.3)	0.38 (0.09–1.67)	0.200		
Hematologic malignancy	44 (19.9)	10.45 (3.78–29.07)	< 0.001	20.97 (5.01–87.82)	< 0.001
Liver transplantation	15 (6.8)	2.58 (0.73–9.06)	0.141	40.08 (4.87–329.76)	0.001
Neutropenia	42 (19.0)	8.86 (3.21–24.42)	< 0.001		
CCI ≥ 3	123 (55.7)	1.25 (0.46–3.37)	0.660		
Invasive procedure/ICU					
Central venous catheter	124 (56.1)	3.04 (0.98–9.43)	0.055		
Artificial airway	57 (25.8)	0.92 (0.33–2.73)	0.945		
Intra-abdominal surgery	21 (9.5)	1.86 (0.53–6.55)	0.332		
Parenteral nutrition	114 (51.6)	1.82 (0.66–5.03)	0.246		
ICU Stay	157 (71.0)	1.59 (0.51–4.95)	0.424		
Previous antibiotic use					
Ampicillin	29 (13.1)	0.31 (0.04–2.38)	0.261		
Glycopeptide	121 (54.8)	6.39 (1.45–28.17)	0.014		
3rd- or 4th-generation cephalosporin	84 (38.0)	1.26 (0.47–3.37)	0.642		
Carbapenem	123 (55.7)	4.35 (1.23–15.36)	0.022		
Piperacillin/tazobactam	132 (59.7)	1.04 (0.36–2.77)	0.994		
Metronidazole	33 (14.9)	2.40 (0.83–6.91)	0.105		
Fluoroquinolone	68 (30.8)	0.48 (0.14–1.69)	0.221		
Aminoglycoside	20 (9.0)	1.19 (0.27–5.25)	0.816		
Linezolid	16 (7.2)	2.61 (0.74–9.17)	0.134		

Data presented as numbers (%) unless indicated otherwise. BMI, body mass index; LC, liver cirrhosis; AKI, acute kidney injury; RRT, Renal replacement therapy, CCI, Charlson comorbidity index; ICU, intensive care unit

The time between rectal VREF detection and the initial day of blood-culture positivity was not statistically different between ST17 (median, 8.5 days; range, 4–139 days) and non-ST17 cohorts (median, 38.5 days; range, 3–187 days) (*P* = 0.181).

### Genetic relatedness between rectal and blood VREF isolates

**Fig. 2:**
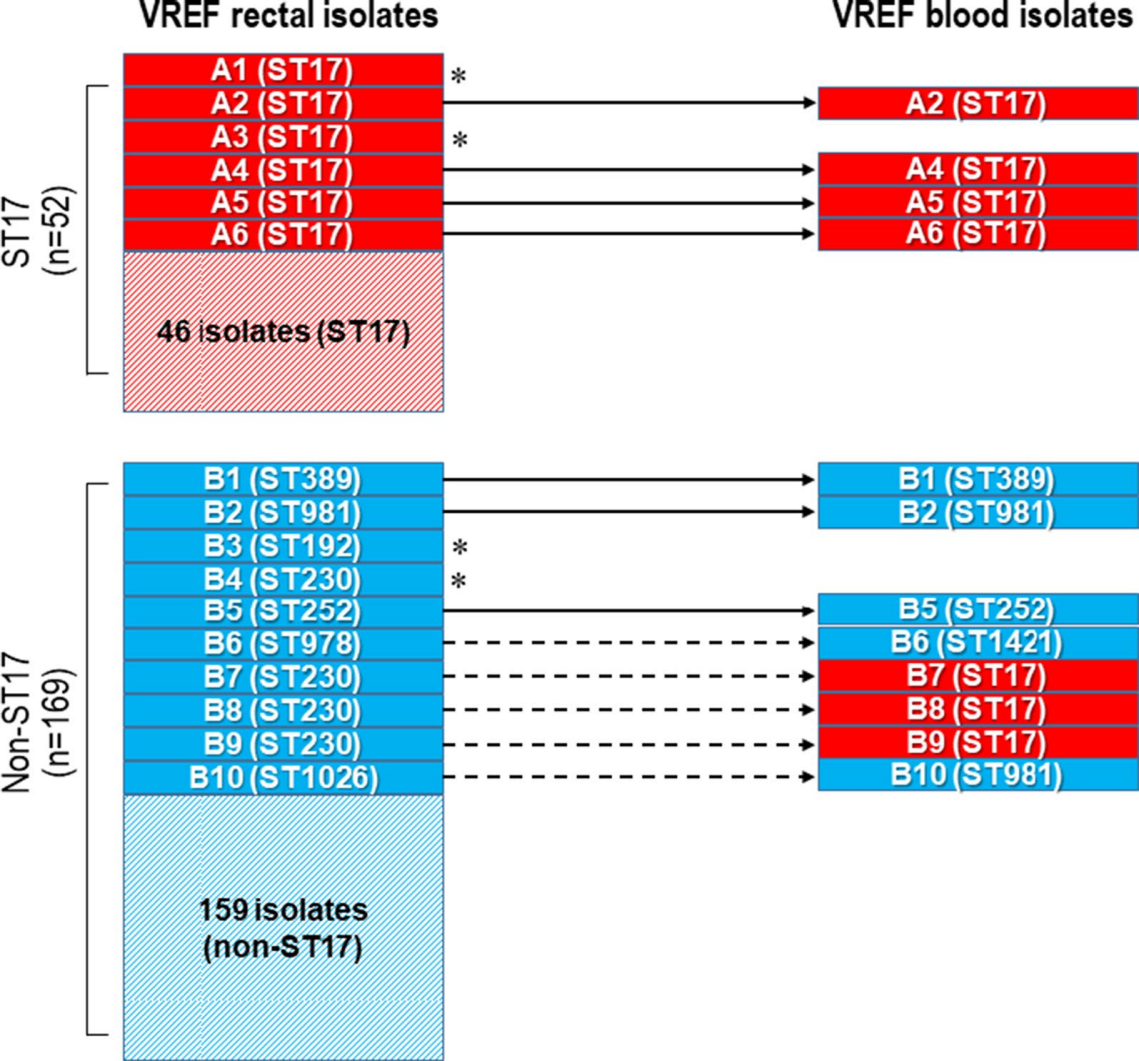
Comparison of sequence types between rectal and blood isolates from patients carrying vancomycin-resistant *Enterococcus faecium* (VREF) and developing subsequent bacteremia. Solid box, cases developing subsequent bacteremia; hatched box, cases not developing bacteremia; red, ST17; blue, non-ST17; solid arrow, identical sequence types; dotted arrow, non-identical line arrow; asterisk, cases of which blood isolates were not stored

Of the 16 patients who developed subsequent VREF bacteremia, 12 VREF blood isolates were available for microbiological analysis. The ST distribution of blood VREF isolates was different from that of rectal isolates (Fig. 2). ST17 was the most common genotype (58.3%) among blood VREF isolates. Comparison of STs between rectal and blood VREF isolates showed that only 7 of 12 cases (58.3%) had identical STs. Among 7 cases with identical STs between rectal and blood isolates, 4 belonged to ST17. In the ST17 VREF carriers who developed subsequent bacteremia, all cases were caused by ST17, but in the non-ST17 carriers, subsequent bacteremia was often caused by another ST, including ST17 (Fig. 2). Among 7 VREF pairs with identical STs between rectal and blood isolates, 6 pairs showed identical PFGE patterns and 1 pair showed a 88.9% similarity (Additional file 1: Fig. S3).

## Discussion

CC17 has been frequently reported as a major genotype causing healthcare-associated outbreaks. CC17 is also related to antibiotic resistance and the carriage of virulence factors such as *esp* and *hyl* [14, 27]. Our study revealed that carriage of ST17 VREF was significantly associated with a risk of developing subsequent VREF bacteremia. In multivariate analyses using 2 models, carriage of ST17 VREF had a 4.02- or 7.14-fold higher risk of developing subsequent VREF bacteremia compared with carriage of non-ST17 VREF. Although ST17 VREF had more virulence factors compared with non-ST17 VREF in this study, *esp* or *hyl* were not risk factors for subsequent VREF bacteremia. Our previous unpublished one-year study in 2014 on bacteremia also showed ST17 was the most frequent ST among both VREF (36.4%, 12/33) and and vancomycin-susceptible *E. faecium* (17.6%, 13/74) bacteremia. Further studies on virulence determinants of ST17 VREF would be needed.

The underlying conditions of liver transplantation and hematologic malignancy were strongly associated with a higher incidence of subsequent VREF bacteremia. Prior studies reported that approximately 30% of VRE-colonized patients with hematologic malignancy or patients who had received a liver transplant developed subsequent VRE bacteremia [28, 29], but no genotypic characterization was reported. Our study was strengthened by analysis of these risk factors through a multivariate analysis that included genotype. In our study, rates of subsequent bacteremia were slightly lower compared with previous studies (22.7% of patients with hematologic malignancy, 20% of patients who had undergone liver transplantation).

Our finding that some patients carrying non-ST17 VREF developed subsequent bacteremia by ST-17 VREF, although all cases in which ST-17 VREF carriers who developed subsequent bacteremia were caused by ST17 VREF, suggests that the ST17 clone has a higher virulence. We also demonstrated that ST17 VREF exhibits strong genetic concordance between rectal and blood VREF isolates in patients who developed subsequent bacteremia.

There are some limitations in our study. First, because the clinical data were collected retrospectively, some data may be incorrect or missing. Second, caution should be taken in generalizing our findings, given VREF colonizers in our cohort were only identified through active surveillance by rectal swabs among the targeted high-risk population in a single center. Third, blood isolates were collected, and molecular analysis was conducted, in only 75% of patients who developed subsequent bacteremia. Despite these limitations, and to our knowledge, this is the first study to reveal a specific genotype as a risk factor of subsequent VREF bacteremia among hospitalized patients carrying VREF.

## Conclusions

The ST17 genotype is associated with occurrence of subsequent VREF bacteremia among hospitalized patients carrying VREF. Patients with hematologic malignancy and those receiving liver transplants are also at high risk of developing subsequent VREF bacteremia. Based on our results, VRE active screening for hospitalized patients can be limited to high-risk groups of bacteremia. Such a targeted infection control strategy can reduce the cost and time for VRE active surveillance for hospitalized patients.

## Supplementary Information

**Additional file 1**.** Supplementary Table 1**. Sequence type and virulent factor of rectal vancomycin-resistant* Enterococcus faecium* isolates.** Supplementary Fig. 1**. Study population included in the study.** Supplementary Fig. 2**. E-burst diagram showing sequence type (ST) distribution of rectal vancomycin-resistant* Enterococcus feacium* (VREF) isolates.** Supplementary Fig. 3**. Dendrogram of the pulsed field gel electrophoresis patterns of 7 vancomycin-resistant* Enterococcus feacium* (VREF) pairs with identical sequence types (STs) between rectal and blood isolates.

## Data Availability

The datasets generated and/or analyzed during the current study are available from the corresponding author on reasonable request.
