# Supplementary material for: Sequence type 17 is a predictor of subsequent bacteremia in vancomycin-resistant Enterococcus faecium-colonized patients: a retrospective cohort study
Source: Antimicrob Resist Infect Control. 2021 Jul 22;10:108. doi: 10.1186/s13756-021-00980-1 (PMC8299594; doi:10.1186/s13756-021-00980-1)

**Supplementary Table 1 Sequence type and virulent factor of rectal vancomycin-resistant *Enterococcus faecium* isolates**

| Cohorts | MLST | | Virulence factors | | | |
| --- | --- | --- | --- | --- | --- | --- |
|  | **Relation to ST17** | **Sequence type** | ***esp* + *hyl* +** | ***esp* + *hyl* -** | ***esp* - *hyl* +** | ***esp* - *hyl* -** |
| Cohort  ST17  (52) | **ST17 (52)** | ST17 (52) | 51 | 0 | 0 | 1 |
| Cohort  non-ST17  (169) | Single-locus variants  (32) | ST78 (13) | 9 | 1 | 3 | 0 |
|  |  | ST117 (1) | 0 | 0 | 0 | 1 |
|  |  | ST202 (3) | 3 | 0 | 0 | 0 |
|  |  | ST252 (2) | 0 | 0 | 2 | 0 |
|  |  | ST789 (8) | 6 | 0 | 0 | 2 |
|  |  | ST980 (2) | 2 | 0 | 0 | 0 |
|  |  | ST1061 (1) | 1 | 0 | 0 | 0 |
|  |  | ST1065 (1) | 1 | 0 | 0 | 0 |
|  |  | ST1066 (1) | 1 | 0 | 0 | 0 |
|  | Double-locus variants  (77) | ST18 (2) | 1 | 0 | 0 | 1 |
|  |  | ST80 (4) | 0 | 1 | 1 | 2 |
|  |  | ST132 (2) | 1 | 0 | 1 | 0 |
|  |  | ST192 (12) | 12 | 0 | 0 | 0 |
|  |  | ST230 (35) | 33 | 0 | 2 | 0 |
|  |  | ST260 (4) | 0 | 0 | 0 | 4 |
|  |  | ST359 (1) | 1 | 0 | 0 | 0 |
|  |  | ST389 (4) | 2 | 0 | 2 | 0 |
|  |  | ST410 (1) | 1 | 0 | 0 | 0 |
|  |  | ST856 (1) | 0 | 0 | 1 | 0 |
|  |  | ST974 (1) | 0 | 0 | 0 | 1 |
|  |  | ST988 (2) | 1 | 0 | 1 | 0 |
|  |  | ST1062 (4) | 4 | 0 | 0 | 0 |
|  |  | ST1063 (1) | 1 | 0 | 0 | 0 |
|  |  | ST1064 (1) | 1 | 0 | 0 | 0 |
|  |  | ST1068 (1) | 0 | 0 | 1 | 0 |
|  |  | ST1078 (1) | 1 | 0 | 0 | 0 |
|  | Others  (60) | ST547 (3) | 3 | 0 | 0 | 0 |
|  |  | ST778 (3) | 3 | 0 | 0 | 0 |
|  |  | ST787 (1) | 1 | 0 | 0 | 0 |
|  |  | ST927 (9) | 9 | 0 | 0 | 0 |
|  |  | ST973 (1) | 0 | 0 | 1 | 0 |
|  |  | ST975 (1) | 0 | 0 | 0 | 1 |
|  |  | ST978 (1) | 1 | 0 | 0 | 0 |
|  |  | ST979 (3) | 3 | 0 | 0 | 0 |
|  |  | ST1067 (1) | 1 | 0 | 0 | 0 |
|  |  | ST1070 (2) | 2 | 0 | 0 | 0 |
|  |  | ST1071 (1) | 0 | 0 | 1 | 0 |
|  |  | ST1072 (1) | 1 | 0 | 0 | 0 |
|  |  | ST1077 (1) | 0 | 0 | 1 | 0 |
|  |  | ST972 (1) | 1 | 0 | 0 | 0 |
|  |  | ST976 (2) | 0 | 0 | 0 | 2 |
|  |  | ST981 (17) | 17 | 0 | 0 | 0 |
|  |  | ST1026 (6) | 1 | 0 | 5 | 0 |
|  |  | ST1069 (1) | 1 | 0 | 0 | 0 |
|  |  | ST1073 (1) | 1 | 0 | 0 | 0 |
|  |  | ST1075 (1) | 0 | 0 | 1 | 0 |
|  |  | ST1074 (1) | 1 | 0 | 0 | 0 |
|  |  | ST1076 (2) | 0 | 0 | 2 | 0 |
|  |  | | 179 | 2 | 25 | 15 |

Numbers in parentheses are the number of strains.

**Supplementary Fig. 1 Study population included in the study**
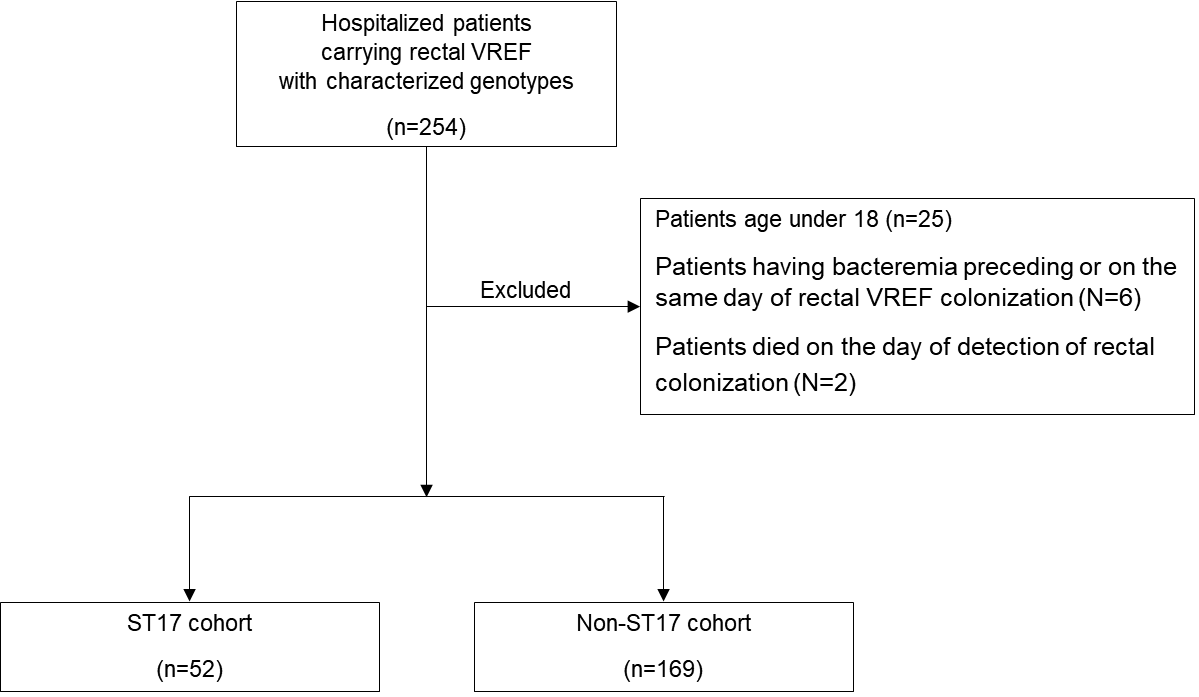


VREF, vancomycin-resistant *Enterococcus feacium.*

**Supplementary Fig. 2 E-burst diagram showing sequence type (ST) distribution of rectal vancomycin-resistant *Enterococcus feacium* (VREF) isolates.**


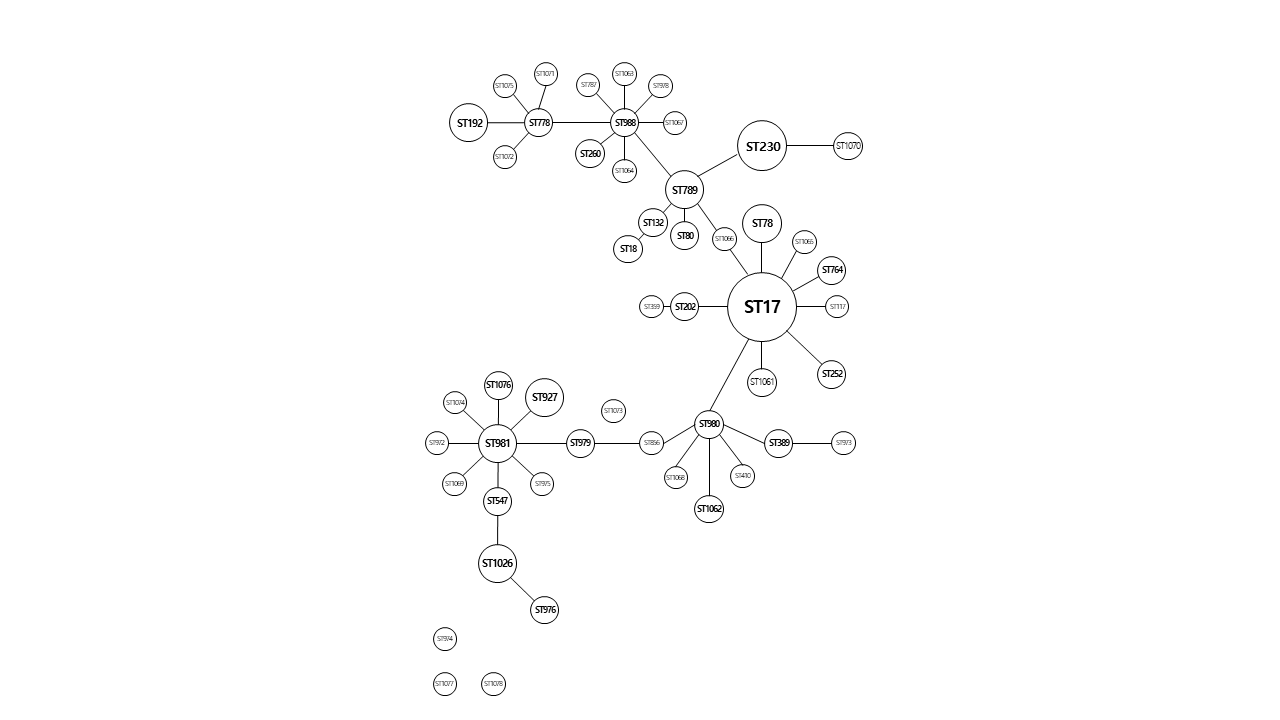


**Supplementary Fig. 3 Dendrogram of the pulsed field gel electrophoresis patterns of 7 vancomycin-resistant *Enterococcus feacium* (VREF) pairs with identical sequence types (STs) between rectal and blood isolates.**


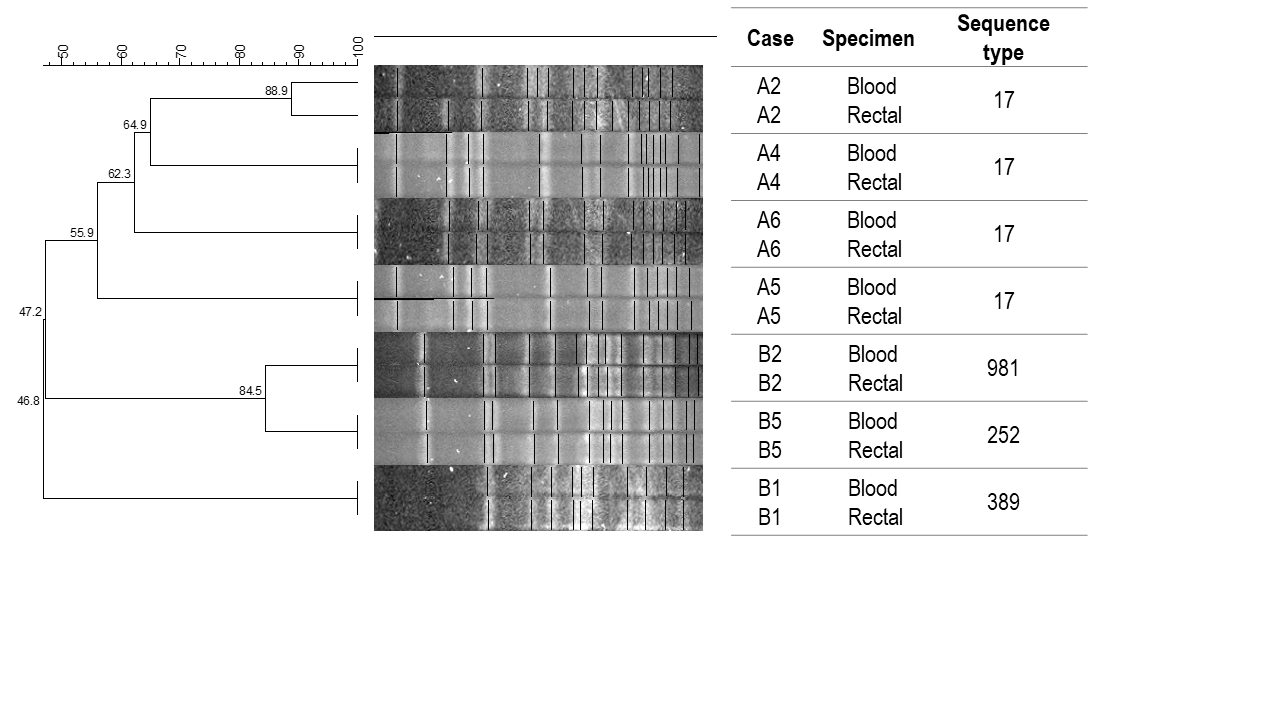

Supplement: Supplementary file 1 — Additional file 1. Supplementary Table 1. Sequence type and virulent factor of rectal vancomycin-resistant Enterococcus faecium isolates. Supplementary Fig. 1. Study population included in the study. Supplementary Fig. 2. E-burst diagram showing sequence type (ST) distribution of rectal vancomycin-resistant Enterococcus feacium (VREF) isolates. Supplementary Fig. 3. Dendrogram of the pulsed field gel electrophoresis patterns of 7 vancomycin-resistant Enterococcus feacium (VREF) pairs with identical sequence types (STs) between rectal and blood isolates. [file 13756_2021_980_MOESM1_ESM.docx]
